# Mapping the Complexity of Suicide by Combining Participatory Modeling and Network Science

**DOI:** 10.1145/3487351.3488271

**Published:** 2022-01-20

**Authors:** Philippe J. Giabbanelli, Michael C. Galgoczy, Duc M. Nguyen, Romain Foy, Ketra L. Rice, Nisha Nataraj, Margaret M. Brown, Christopher R. Harper

**Affiliations:** Department of Computer Science & Software Engineering, Miami University, Oxford, OH, United States; Department of Computer Science & Software Engineering, Miami University, Oxford, OH, United States; Department of Computer Science & Software Engineering, Miami University, Oxford, OH, United States; IMT Mines Alés, Institut Mines-Telecom, Alés, France; National Center for Injury Prevention and Control, Centers for Disease Control and Prevention (CDC), Atlanta, GA, United States; National Center for Injury Prevention and Control, Centers for Disease Control and Prevention (CDC), Atlanta, GA, United States; National Center for Injury Prevention and Control, Centers for Disease Control and Prevention (CDC), Atlanta, GA, United States; National Center for Injury Prevention and Control, Centers for Disease Control and Prevention (CDC), Atlanta, GA, United States

**Keywords:** Knowledge Map, Suicide, Participatory Model

## Abstract

Suicide rates are steadily increasing among youth in the USA. Although several theories and frameworks of suicide have been developed, they do not account for some of the features that define suicide as a complex problem, such as a large number of interrelationships and cycles. In this paper, we create the first c omprehensive m ap o f a dverse c hildhood experiences (ACEs) and suicide for youth, by combining a participatory approach (involving 15 subject-matter experts) and network science. This results in a map of 946 edges and 361 concepts, in which we identify ACEs to be the most important factor (per degree centrality). The map is openly shared with the community to support further network analyses (e.g., decomposition into clusters). Similarly to the high-impact Foresight Map developed in the context of obesity, the largest map on suicide and ACEs to date presented in this paper can start a discussion at the crossroad of suicide research and network science, thus bringing new means to address a complex public health challenge.

To address the public health challenge of steadily increasing suicide rates among youth in the USA [1], it is essential to understand what drives the increase in youth suicide ideation and implement interventions to reduce ideation and prevent attempts. These objectives are challenging given the complexity of suicide, as it is rarely caused by a single factor. Although suicide has long been recognized as a multifactoral issue [2, 3], there is still limited understanding regarding the complex relationships between the factors contributing to, or affected by, intentional self-harm. Ideation-to-action theories of suicide [4, 5] and the social ecological model [6] have enabled important advances to suicidal behaviors (e.g., based on burdensomeness and belongingness, or a threshold of tolerance to life stressors) and continue to provide the foundational framework for modeling studies [7]. However, these theories and frameworks do not cover several of the features that define complex problems such as suicide: nonlinearity (e.g., cumulative trauma grows faster than the number of adverse experiences), large number of ties or ‘interrelationships’ between concepts (e.g., across personal, family, community, and societal domains), and loops/cycles (i.e. when a change in one factor eventually impacts the same factor).

In this paper, we develop a network representation or ‘system map’ of suicide for youth, with the objectives of accounting for a large number of factors and the many loops involved in this problem. This map covers both suicide and ACEs, thus integrating notions that have previously been mapped in isolation. The map thus contributes to the needs for more comprehensive tools guiding suicide prevention planning [8].

The design of large system maps for complex problems typically faces one major obstacle: data may come in different types (e.g. qualitative, quantitative risk ratios or odds ratios) and from various sources (e.g. longitudinal studies, meta-reviews). While creating a model from a single type and source of data simplifies the assessment of its validity, our study demonstrates that the fragmentation of data would drastically limit the content of such a model. Consequently, this multidisciplinary study uses mixed-methods to achieve a comprehensive system map by combining network science with participatory modeling during one-on-one mapping interviews with 15 Subject Matter Experts (SMEs).

In section I, we explain our steps: identifying and inviting SMEs, structuring an individual map through one-on-one interviews, assembling the maps into a single one, and simplifying it. Section II summarizes the characteristic of the network which we have built and openly shared at https://osf.io/7nxp4/.Finally, section III briefly covers the potential uses of this network for policymaking and network mining.

## I. Methods

### A. Identification and Invitation of Subject Matter Experts

Systematic approaches to identify participants in a participatory modeling study include the application of selection criteria, nomination by a committee, and referral by the participants (i.e. ‘snowball sampling’). We use these three strategies as follows. Selection criteria for the SMEs included having an established track record in research and public health prevention of suicide and/or ACEs and/or clinical expertise in the therapy and treatment of youth presenting suicidal behavior. The inclusion of therapists, clinical practitioners, and public health experts is essential to obtain a comprehensive coverage of risks and protective factors for suicide, which include but are not limited to ACEs. Our participants were experts within (*n* = 10) and outside the CDC (*n* = 5). A track record consists of graduate-level training (e.g., MS, MPH, PhD) and a minimum of 6 years of experience either in research or practice. Based on these criteria, a committee created a purposeful sample of SMEs to ensure we would engage with participants who could communicate experiences and expertise related to suicide in a comprehensive and reflective manner, and who were available and willing to participate within the study time frame. The committee included CDC experts from the National Center for Injury Prevention and Control’s (NCIPC) Division of Injury Prevention Suicide Prevention Team and Division of Violence Prevention Child Abuse Neglect and Adversity Team. Given the cross-sectoral nature of ACEs and suicide risks or preventative factors, the committee assembled an interdisciplinary team of SMEs including behavioral scientists, health scientists and epidemiologists, and physicians. Referrals from participating SMEs served to recruit additional colleagues across other divisions within NCIPC and outside the CDC (i.e. SMEs from other federal agencies, non-profit organizations, private practice, and universities). After each SME was identified, an email invitation was sent with proposed calendar dates within the next two weeks for scheduling. The study protocol was examined given the CDC guidelines to determine the need for approval by the Institutional Review Board (IRB) and Office of Management and Budget (OMB). The study received exemption from both.

### B. One-on-one interviews

Before conducting an individual interview, a mapping approach requires an identification of the problem of interest and a definition of the system’s boundaries [9, 10]. The problem must be sufficiently isolated to avoid ambiguities, which can lead to seemingly contradictory answers from the SMEs and raise issues in data analysis. Asking “what causes suicide?” would potentially be ambiguous as SMEs may think of different stages, from suicidal thoughts to suicide attempt and completion. In line with other models [11], we thus decomposed suicide into four problems for the suicide SMEs: suicide ideation, suicide planning, suicide attempt, and suicide fatality. Each interview is conducted and recorded using WebEx video conferencing after receiving informed consent from the SME. The interview starts with a description of our project, which also serves as an opportunity to reiterate the system boundaries. SMEs are asked if they have any questions, then the interview proceeds with eliciting the map.

We begin with the problem of interest and systematically ask the participant for causes and consequences, while clarifying whether the causal link (i.e., edge) they mentioned leads to an increase (e.g., internalized trauma increases the risk for suicide ideation) or a decrease (e.g., connectedness lowers the risk of suicide ideation). We do not ask confirmatory questions (e.g., “do you think homelessness increases suicide ideation?”), as they may bias an SME in endorsing an idea. The list of factors is frequently read back to the SME such that they can identify any additional ones. Once the proximal causes and consequences are identified, we shift into a second phase to identify mediators, more distal factors, or interrelationships. Questions include identifying how causal links work, particularly where there appears to be a logical gap; forming connections between existing factors; and identifying additional causes.

As case studies have abundantly demonstrated that cognitive limitations prevent stakeholders from identifying loops [12], we actively monitor the structure of the network as the interview unfolds. The network is approximately structured by the modeler during the discussion using MentalModeler to track what has been said and prepare the next questions.

### C. Transcribing an interview into an individual map

**Fig. 1.**
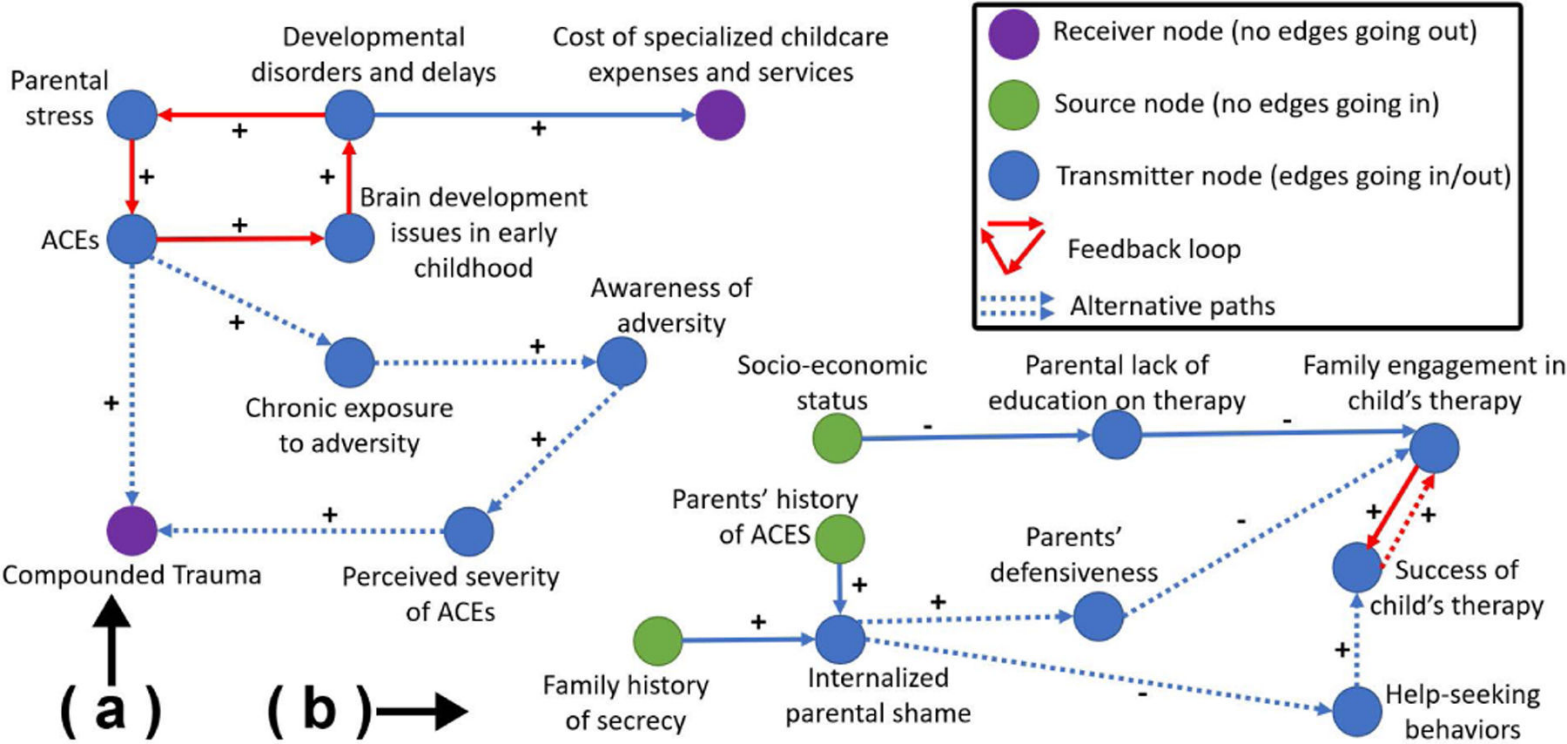
A map can be analyzed with respect to its type of nodes (receivers, sources, transmitters) or the existence of structures involving multiple edges (e.g., feedback loops, alternative paths).

We use the systematic method from Kim and Andersen to transform each recorded interview into a system map [13]. We identify the concepts in the discussion (e.g., homelessness, suicide ideation) and track their causal connections (e.g., homelessness increases the risk for suicide ideation). In a system map, concepts are represented as nodes while connections are represented as typed directed edges. In other words, each part of the interview is transformed into a schematic description of a cause variable, an effect variable, and a relationship polarity. After each interview has been transformed into a map, we examine the structure of the map for validation with respect to: ① **Node types**. As shown in Figure 1, causal impacts can end at a node (*receiver*; typically used as output), start at a node (*source*; typically used as parameter), or pass through a node (*transmitter*). Although an early model may have a lot of parameters, we expect this to be reduced in more elaborate models as SMEs start to capture that such parameters are in fact partly driven by other concepts. ② **Diameter**. When sequences of causal links tend to be short (e.g., *A* → *B* → *C*), there is a risk that the arguments have not been well elaborated [14]. ③ **Cycles**. There are feedback loops in suicide [11, 15], but cognitive limitations may prevent SMEs from identifying such loops [12]. A map created with a trained facilitator should thus exhibit loops. ⑤ **Density**. The notion captures the overall level of interconnectedness in the map. Generally, we expect very sparse maps with very low ratios [16], since participants are selective in the causal connections that they perceive. As maps cover more of the problem space (e.g. larger diameter), this ratio decreases.

### D. Assembling individual maps into a single map

Combining all individual maps into one is a challenge due to variations in language across the SMEs. Some studies prevent this problem from appearing by limiting the SMEs to only designate concepts from a pre-established list [17], possibly including ‘distractors’ or ‘misleading’ concepts to ensure careful selection [18]. However, such limitations may force SMEs to artificially think alike and prevent the emergence of concepts that were not previously considered [19]. After adding all causal links and concepts across individual maps into a single one, we manually identify all equivalent forms of a concept and resolve them into a single term. Five of the authors (including a suicide SME and an ACE SME) thus identify and reduce variations in language iteratively, until each concept appears through a unique term. Variations are tracked and documented in a ‘thesaurus’, available on our repository (https://osf.io/7nxp4/). The thesaurus can be reused by other researchers needing to reduce variability in similar qualitative inquiries featuring open-ended coding schemes.

Based on previous studies, the first aggregate map of a complex system can be ‘large and unwieldy’ [20] thus it needs to undergo a simplification process. We simplify the model by a combination of (i) removing some of the source and receiver concepts (i.e. reducing the number of model parameters and outputs) based on consultation with SMEs, and (ii) simplifying long chains of causations such that all parts of the model have a comparable level of granularity. As an unfiltered aggregate map can be challenging to navigate, we automatize parts of this process using network science tools (provided as scripts on https://osf.io/7nxp4/) to (i) extract all source and receiver concepts as well as (ii) identify concepts that act as intermediate on a path and may thus be skipped.

## II. Results

### A. Map Building Process

A total of 17 invitations were emailed, with 15 affirmative responses (listed in the acknowledgments), and 2 non-responses. The fifteen SMEs accepting to participate were interviewed for the study between June 29, 2020 and July 21, 2020. Interviews lasted 55:1 ± 7.4 minutes. Participants aptly saw the system as being composed of loops, rather than as a list of determinants. Rather than focusing on direct causes or consequences, participants accounted for distal factors as evidenced by an average diameter of 8. On average, 15 out of the 85 causal links are concentrated around a single factor (i.e. max degree), which indicates a small level of centralization.

When combining the 15 individual maps into a single one, we found that 131 terms appeared in several maps under different names. These differences were resolved by adopting a single name (e.g., “racism” instead of “structural racism” and “racial injustice”). Each of the 131 terms had an average of 4 linguistic variations. Eight concepts had over ten linguistic variations, which indicates their importance across interviews: mental health disorders, connectedness, coping skills, family financial stress, economic policies for ACEs, parents’ substance use, protective factors, and child abuse or neglect.

### B. Map

Characteristics of the map after simplification are presented in Table 1. We note its total of 946 edges and 361 concepts, which makes it the largest map on ACEs and suicide [21]. There are only two receiver nodes (Community exposure to suicide, Involvement in violence), which stand for consequences that go ‘beyond’ the life of a child or adolescent. The most important factor in the map in terms of degree are Adverse Childhood Experiences (ACEs). It has a total of 92 antecedents and consequents, in part because ACEs encompass a large number of forms of abuse.

### III. Discussion

We developed the largest system map to date on suicide and Adverse Childhood Experiences (ACEs), totaling 361 concepts and 946 interrelationships. As shown by the many steps of our methodology, the development of a comprehensive system map is a large undertaking. However, similar to how the Foresight Obesity Map shaped conversations and guided numerous efforts in obesity research [22], the investment that we made in developing a system map on ACES and suicide among youth provides a decision-support tool that can benefit researchers and public health practitioners in ACEs and youth suicide prevention. The case of the Foresight Obesity Map exemplifies how numerous future studies can be derived from network analyses of the map itself, its comparison with data sources, or minor alterations to our process. For instance, a dedicated study examined the structure of the Foresight Obesity Map from a network perspective by reducing it to core ‘groups’ or components [23] and their implications for solutions. In another case, the authors compared the map developed from subject-matter experts (as built here) with another one, developed by members of the community who experience the issues covered by the map [24]. As suicide is an interdisciplinary problem, a comparison of maps built by sizeable subgroups can serve to investigate potential expert biases (e.g., from training or practice) toward the importance of their own field over others [24]. In sum, there are numerous instances in which the release of a comprehensive map [25] is followed by an extensive analysis [26] with implications for the field. The development of our map and its public release thus provide the first step in a larger vision of using network science methods in suicide research to more effectively guide data collection and cope with the complexity of the problem.

## Figures and Tables

**TABLE I:** Characteristics of the whole system map.

Structural characteristic	Value
Number of nodes	361
Number of source nodes	113
Number of receiver nodes	2
Number of edges	946
Density	0.007
Average degree	5.240
Maximum degree	92 (ACEs)
Diameter	12
